# Retracted: Study of Effect of Salvianolic Acid B on Motor Function Recovery in Rats with Spinal Cord Injury

**DOI:** 10.1155/2017/8234878

**Published:** 2017-09-24

**Authors:** BioMed Research International

BioMed Research International has retracted the article titled “Study of Effect of Salvianolic Acid B on Motor Function Recovery in Rats with Spinal Cord Injury” [1]. The article was found to contain a substantial amount of material from the following published article: “Xiao-bin Bi, Yu-bin Deng, Dan-hui Gan and Ya-zhu Wang, (2008), Salvianolic acid B promotes survival of transplanted mesenchymal stem cells in spinal cord-injured rats. Acta Pharmacologica Sinica, 29: 169–176. doi: 10.1111/j.1745 7254.2008.00710.x.” Additionally, Figure 1 in this article [1] is the same as Figure 1 in the 2008 article, but it shows signs of manipulation.
